# MicroRNA-208a Dysregulates Apoptosis Genes Expression and Promotes Cardiomyocyte Apoptosis during Ischemia and Its Silencing Improves Cardiac Function after Myocardial Infarction

**DOI:** 10.1155/2015/479123

**Published:** 2015-11-25

**Authors:** Hasahya Tony, Kai Meng, Bangwei Wu, Aijia Yu, Qiutang Zeng, Kunwu Yu, Yucheng Zhong

**Affiliations:** ^1^Laboratory of Cardiovascular Immunology, Institute of Cardiology, Union Hospital, Tongji Medical College, Huazhong University of Science and Technology, 1277 Jie-Fang Avenue, Wuhan 430022, China; ^2^Department of Cardiology, The Second Hospital of Shandong University, Jinan, Shandong 250033, China; ^3^Department of Cardiology, Huashan Hospital, Shanghai Medical College, Fudan University, Shanghai 200040, China; ^4^Department of Ultrasound, Union Hospital, Tongji Medical College, Huazhong University of Science and Technology, 1277 Jie-Fang Avenue, Wuhan 430022, China

## Abstract

*Aims*. miR-208a is associated with adverse outcomes in several cardiac pathologies known to have increased apoptosis, including myocardial infarction (MI). We investigated if miR-208a has proapoptotic effects on ischemic cardiomyocytes and if its silencing has therapeutic benefits in MI.* Methods and Results*. The effect of miR-208a on apoptosis during ischemia was studied in cultured neonatal mice myocytes transfected with agomir or antagomir. Differential gene expression was assessed using microarrays. MI was induced in male C57BL/6 mice randomly assigned to antagomir (*n* = 6) or control group (*n* = 7), while sham group (*n* = 7) had sham operation done. Antagomir group received miR208a antagomir, while control and sham group mice received vehicle only. At 7 and 28 days, echocardiography was done and thereafter hearts were harvested for analysis of apoptosis by TUNEL method, fibrosis using Masson's trichrome, and hypertrophy using hematoxylin and eosin. miR-208a altered apoptosis genes expression and increased apoptosis in ischemic cardiomyocytes. Therapeutic inhibition of miR-208a decreased cardiac fibrosis, hypertrophy, and apoptosis and significantly improved cardiac function 28 days after MI.* Conclusion*. miR-208a alters apoptosis genes expression and promotes apoptosis in ischemic cardiomyocytes, and its silencing attenuates apoptosis, fibrosis, and hypertrophy after MI, with significant improvement in cardiac function.

## 1. Introduction

Apoptosis, a distinct form of cell death, is at the heart of both mechanical and molecular mechanisms of cardiomyocyte loss during ischemia. After MI, there is heightened apoptosis in the peri-infarct and noninfarcted myocardium, and this coincides with increased left ventricular diameter and decreased cardiac function [1]. To this effect, therapies that inhibit apoptosis in MI have been shown to improve cardiac function [2, 3].

MicroRNAs (miR) are small noncoding ribonucleic acids (RNAs) measuring 18–25 nucleotides that are involved in posttranscription regulation of gene expression [4, 5]. miR-208a is a cardiac specific microRNA coded for in an intron of myosin heavy chain 6 (Myh6) gene and regulates cardiac conduction, stress response, and gene expression [6–9]. Several researches have shown that its inhibition or deletion is associated with downregulation of cardiac fibrosis and hypertrophy in response to various stress stimuli [6–8]. It has also been shown to be dysregulated in several cardiac diseases including myocardial infarction and dilated cardiomyopathy, in which it is associated with adverse outcomes [10, 11]. However, no study as yet has been done to investigate its role in cardiomyocyte apoptosis despite these cardiac pathologies being associated with increased myocyte apoptosis. We therefore investigated the effect of miR-208a on cardiomyocyte apoptosis and apoptosis related genes and if its silencing has any therapeutic benefit in myocardial infarction. We report that miR-208a upregulation alters apoptosis genes' expression and promotes cardiomyocyte apoptosis, while its silencing attenuates apoptosis and improves cardiac function after MI. To the best of our knowledge, our study is the first to explore the antiapoptotic effects of miR-208a silencing and the therapeutic benefits of miR-208a antagomir in myocardial infarction.

## 2. Materials and Methods

### 2.1. Cardiomyocyte Cell Culture, Transfection, and Simulated Ischemia

Neonatal mouse cardiomyocyte isolation and culture were performed according to previously described protocols [12]. Cardiomyocytes were transfected with 0.2 nmols/mL of miR-208a agomir for gain of function or 0.4 nmols/mL of miR-208a antagomir for loss of function, by directly adding the agomir or antagomir formulation to the cell culture medium according to manufacturer's instructions (RiboBio Co. Ltd., Guangzhou, China). For Bax silencing, cardiomyocyte cells were transfected with Bax siRNA (5′-AACAGAUCAUGAAGACAGGGG-3′) using Ribofectamine reagent according to manufacturer's instructions (RiboBio Co. Ltd., Guangzhou, China). After 48 hours, the different treatment groups underwent simulated ischemia for 12 hours by placing the cardiomyocytes in a hypoxia chamber with 5% CO_2_ and 95% N_2_ at 37°C in glucose-free DMEM medium. Sham group myocytes did not undergo simulated ischemia. Myocytes were harvested, fixed, and assayed for apoptosis according to previously described protocol [13]. Myocytes were also analyzed for miR-208a levels after culturing.

### 2.2. Microarray Profiling

Microarray profiling was done on custom-made array chips by a service provider (RiboBio Co., Guangzhou, China). RNA was isolated from cultured control or miR-208a agomir transfected cardiomyocytes, 72 hours after transfection. Differential gene expression between controls and agomir transfected cardiomyocytes was analyzed using permutation analysis of differential expression. Gene clustering was performed with cluster 3.0 and heat map images generated in Java tree view. Gene ontology analysis was done using DAVID online tool [14, 15], and apoptosis pathway analysis was done using Ingenuity Pathway Analysis (http://www.ingenuity.com/).

### 2.3. Animal Procedures

All experiments were conducted according to the Guide For the Care and Use of Laboratory Animals and approved by the Animal Care and Utilization Committee of Union Hospital, Tongji Medical College, Huazhong University of Science and Technology, Wuhan, China. The investigation conformed to the Guide for the Care and Use of Laboratory Animals, published by the US National Institute of Health (2011).

### 2.4. Animals and Delivery of Antagomir

Adult male C57BL/6 mice, 8-week-old, were purchased from Wuhan University Animal Research Center. Mice were anesthetized by an intraperitoneal injection of pentobarbital sodium (50 mg/kg), orally intubated, and connected to a rodent ventilator, and ECG monitoring was done using RM6240 data acquisition system sampling at 1 kHz. A left thoracotomy was performed by a horizontal incision at the third intercostal space and myocardial infarction induced by ligation of the left anterior descending coronary artery (LAD) according to previously described protocol [16] and then randomly assigned to Antagomir group (*n* = 6) or control group (*n* = 7). Sham group mice (*n* = 7) had the same procedure done but without ligation of the LAD. Starting within 2 hours after MI, each antagomir group mouse received a total of 300 nmols of miR-208a antagomir given over 3 consecutive days (days 0, 1, and 2) using 0.2 mLs of normal saline as vehicle, by low pressure tail vein injection, while control and sham group mice each received 0.2 mLs of vehicle only by the same method over the same period of time. Echocardiography was performed at 7 and 28 days, and thereafter animals were euthanized and hearts harvested for further analysis.

### 2.5. Histology

At 28 days, animals were euthanized and hearts harvested, cut transversely into blocks, and fixed in 4% paraformaldehyde. These were then embedded in OCT compound (BHD, UK) and then transversely cut into 5 *μ*m thick cryosections for histological analysis. Two cryosections from each of the blocks were stained with Masson's trichrome for assessment of fibrosis and infarct size. The infarct size was measured as the sum of the epicardial and endocardial scar length, divided by the sum of LV epicardial and endocardial circumferences.

For assessment of fibrosis, we randomly selected 4 fields per slide in the peri-infarct zone (PIZ) or 5 fields per slide in the noninfarct zone (NIZ) and calculated collagen volume fraction (CVF) as the ratio of blue stained (collagen) area to the total tissue area. The collagen-rich border zone of vessels and the scar were excluded from the analysis. Image analysis was done using Image-Pro Plus software version 6.

Cardiomyocyte hypertrophy was assessed by capturing cardiomyocyte images over 5 different fields per slide (40x objective). Using Image-Pro Plus software version 6, we obtained mean cardiomyocyte cross-sectional areas, which were compared between groups.

### 2.6. Apoptosis Assay

At 28 days after MI or after 12 hours of simulated cardiomyocyte ischemia, tissues or cells, respectively, were fixed with 4% paraformaldehyde, and apoptosis assay was done by TUNEL method according to previously described protocol [13].

### 2.7. Quantitative Real-Time PCR

Total RNA was extracted from cells and tissue samples using Trizol reagent (Invitrogen, USA) according to the manufacturer's protocol. cDNA was synthesized from 2 *µ*g of total RNA with Takara's reverse transcription kit (Takara, China), according to manufacturer's instructions in a 25 *µ*L volume including 2 *µ*g total RNA, and reverse transcription primer for mRNA genes, or random primers for U6 rRNA and miR-208a specific primers (Bulge-Loop miRNA qPCR Primers from RiboBio, China). Real-time PCR was carried out with the reagents of a Sybr green I mix (Takara, China) in a 20 *µ*L reaction volume (10 *µ*L Sybr green I mix, 200 mM forward and reverse primer, and 2 *µ*L cDNA template) on an MJ Opticon Monitor chromo4 instrument (Bio-Rad, USA) using the following protocol: 95°C for 20 s; 40 cycles of 95°C for 10 s, 60°C for 20 s, and 70°C for 1 s. Normalization for miR-208a was done using U6 while Bax expression was normalized using GAPDH. U6 and Bulge-Loop miRNA qPCR Primers were from RiboBio, China. The following primer sets were used for BAX and GAPDH:

 BAX:
   
*Forward*: TGTTTGCTGATGGCAACTTC
   
*Reverse*: GATGGTTCTGATCAGCTCGG
 GAPDH:
   
*Forward*: 5′-GCTGGCGCTGAGTACGTCGTGGAGT-3′
   
*Reverse*: 5′-CACAGTCTTCTGGGTGGCAGTGATGG-3′

Data analyses were performed using the 2^−ΔΔCt^ method.

### 2.8. Echocardiography

To assess if therapeutic silencing of miR-208a improves cardiac function in MI, 2-dimensional transthoracic echocardiography studies were performed on 4-5 mice from each group, lightly anesthetized with pentobarbital sodium (25 mg/kg), on days 7 and 28, using a GE Voluson ultrasound system equipped with an 11.5 MHZ high frequency probe. Cardiac imaging was done in a parasternal short-axis view at the level of the papillary muscles to record M-mode and determine fractional shortening (FS) and ejection fraction (EF) which are measures of cardiac function.

### 2.9. Statistical Analysis

Statistical analysis was done using SPSS version 20. Groups were compared using independent Student's *t*-test or one-way ANOVA with the Tukey or Games-Howell post hoc tests as appropriate. Statistical significance was defined as a *p* value <0.05 and values are presented as mean ± SEM.

## 3. Results

### 3.1. miR-208a Antagomir and Agomir Are Effective in Silencing or Upregulating miR-208a, Respectively

**Figure 1:**
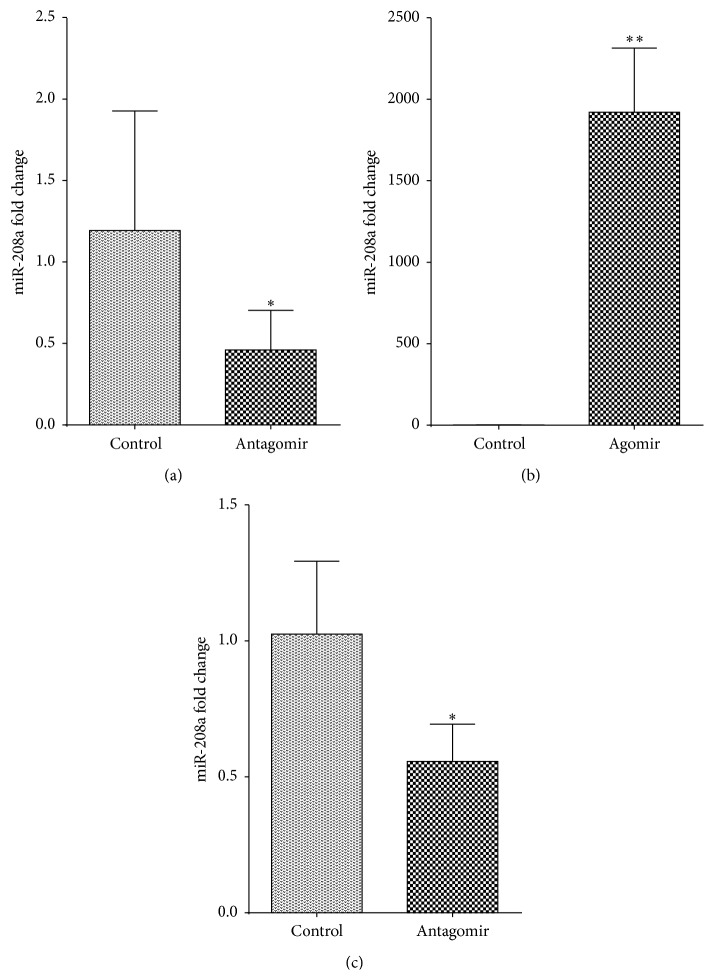
miR-208a agomir and antagomir transfection significantly upregulated or downregulated miR-208a, respectively (^*∗*^ *p* < 0.05, ^*∗∗*^ *p* < 0.001). (a) miR-208a antagomir transfection significantly downregulated miR-208a in cultured neonatal cardiomyocytes 48 hours after transfection (*n* = 4 samples per group). (b) Agomir transfection into neonatal cardiomyocytes significantly upregulated miR-208a (*n* = 4 samples per group). (c) miR-208a antagomir significantly downregulated miR-208a at 28 days after MI (*n* = 4 per group).

Expression of miR-208a in vivo and in vitro was analyzed using quantitative RT-PCR. We found that 0.4 nmol/mL of miR-208a antagomir significantly downregulated miR-208a expression in cultured cardiomyocytes (Figure 1(a)). Similarly, miR-208a agomir upregulated miR-208a expression in neonatal cardiomyocytes 48 hours after transfection (Figure 1(b)). To silence miR-208a, 300 nmols of miR-208a antagomir per mouse was administered after MI, and this significantly downregulated miR-208a expression at 28 days after MI (Figure 1(c)).

### 3.2. miR-208a Alters Apoptosis Genes Expression and Promotes Cardiomyocyte Apoptosis during Ischemia

**Figure 2:**
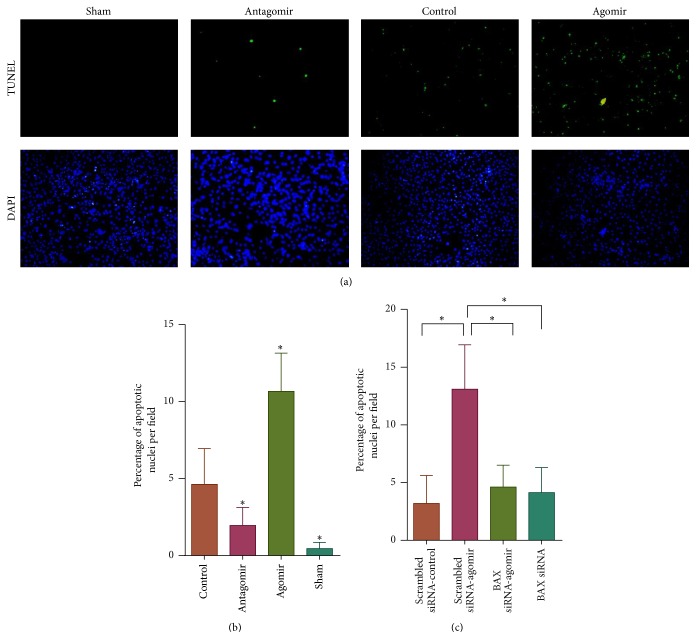
miR-208a upregulation promotes cardiomyocyte apoptosis during ischemia and its silencing attenuates myocyte apoptosis (^*∗*^ *p* < 0.05). (a) Representative TUNEL stained images of neonatal cardiomyocytes showing increased apoptosis following agomir treatment and decreased apoptosis following antagomir treatment and simulated myocyte ischemia (×20 objective). (b) Graph of percentage apoptosis following simulated ischemia of neonatal cardiomyocytes shows that miR-208a agomir significantly upregulated myocyte apoptosis while miR-208a antagomir transfection attenuated ischemia induced apoptosis (*n* = 3 per group). (c) Bax silencing using siRNA attenuated miR-208a agomir induced cardiomyocyte apoptosis during ischemia (*n* = 3 per group).

**Figure 3:**
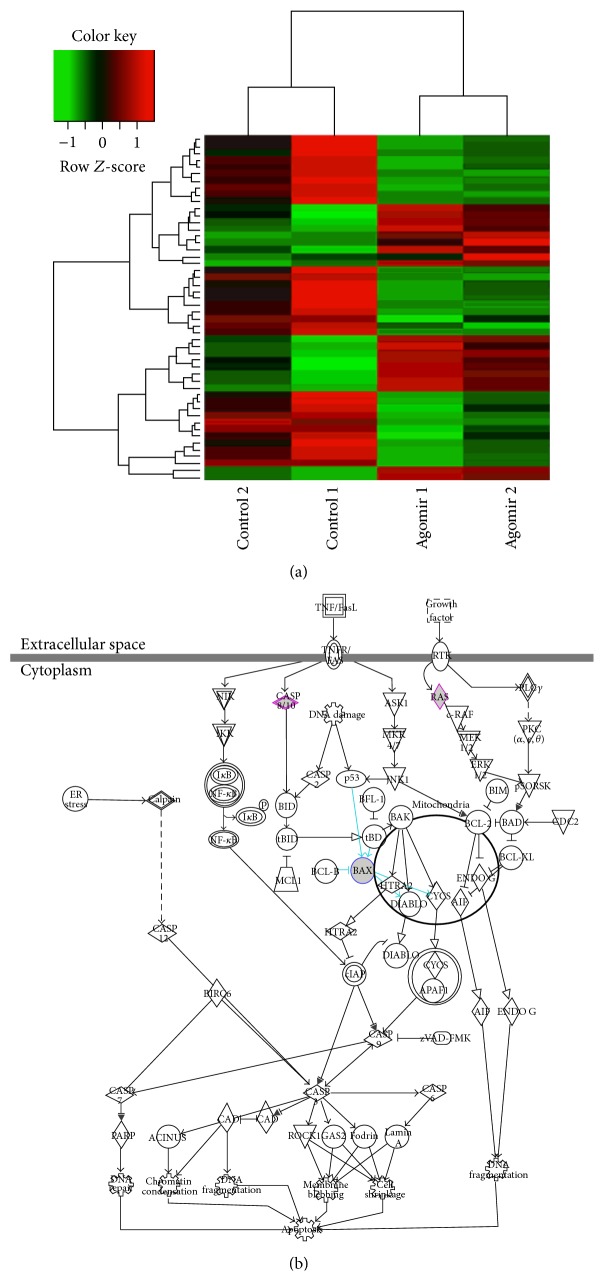
Heat map visualization and pathway analysis of differentially expressed genes. (a) Hierarchical clustering and heat map visualization of the differentially expressed genes after agomir based upregulation of miR-208a. (b) Apoptosis pathway analysis highlighted Bax, Caspase8, and RAS as the miR-208a regulated genes involved in the canonical apoptosis pathways.

Apoptosis, a distinct form of cell death, is a contributor to cardiac dysfunction, remodeling, and heart failure following MI [17, 18]. We investigated the role of miR-208a in cardiomyocyte apoptosis in an ischemic setting. Simulated ischemia of neonatal cardiomyocytes following transfection with miR-208a agomir or antagomir showed that miR-208a upregulation significantly increases apoptosis while its silencing blunts apoptosis during ischemia (Figures 2(a) and 2(b)). To gain insight into the effect of miR-208a on apoptosis related genes, we employed custom-built microarrays (RiboBio Co., Guangzhou, China). Hierarchical clustering and heat map visualization of the differentially expressed genes are shown in Figure 3(a). Cluster analysis of the differentially expressed genes revealed that eight apoptosis related genes were upregulated, while twelve apoptosis related genes were downregulated (Table 1). A closer inspection of the dysregulated genes showed that miR-208a tended to downregulate genes favoring the extrinsic apoptosis pathway, while upregulating those that favor the intrinsic pathway of apoptosis. Analysis of differentially expressed apoptosis genes highlighted Bax, Casp8, and HRAS1 as involved in the canonical apoptosis pathways (Figure 3(b)). Bax is an essential member of the intrinsic pathway of apoptosis. To decipher if Bax was essential for miR-208a induced apoptosis, we used siRNA to silence Bax gene. Bax siRNA attenuated the proapoptotic effect of miR-208a agomir (Figure 2(c)), suggesting that miR-208a functions to promote apoptosis at least in part by upregulating Bax.

### 3.3. miR-208a Silencing Attenuates Apoptosis in MI

**Figure 4:**
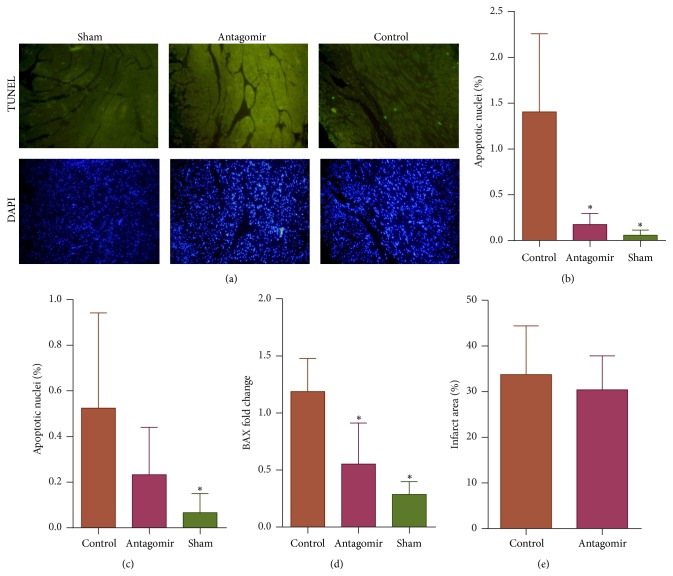
miR-208a silencing attenuates Bax expression and cardiomyocyte apoptosis at 28 days after MI (^*∗*^ *p* < 0.05). (a) Representative tunnel stained images of the peri-infarct area showing that antagomir treatment decreased apoptosis in the peri-infarct area at day 28 (×20 objective). (b) Antagomir treatment significantly attenuated percentage apoptosis in the peri-infarct area (*n* = 3 per group). (c) Bar graph of percentage apoptotic nuclei in the noninfarcted area showing no significant difference between antagomir group and control (*n* = 3). (d) miR-208a antagomir downregulated Bax expression at 28 days after MI (*n* = 4 per group). (e) Although there was a trend towards reduction in infarct size in miR-208a antagomir treated mice compared to controls, this did not reach statistical significance (*n* = 3 per group).

Apoptosis has been observed to occur in the peri-infarct and noninfarcted myocardium and is a contributor to cardiac dysfunction, remodeling, and heart failure following MI [17, 18]. To investigate the antiapoptotic effects of miR-208a silencing in an MI setting, we treated mice with 300 nmols of miR-208a antagomir. Antagomir attenuated peri-infarct apoptosis at 28 days, a finding that is novel and may have therapeutic significance (Figures 4(a) and 4(b)). In the noninfarct area, however, the difference in apoptosis between antagomir and control group hearts only showed a trend towards reduction but did not reach statistical significance (Figure 4(c)). qPCR showed that miR-208a silencing in MI also decreased Bax levels at 28 days (Figure 4(d)).

A comparison of infarct area sizes between control and antagomir groups showed a trend towards reduction in infarct size by antagomir, but this did not reach statistical significance (Figure 4(e)).

### 3.4. miR-208a Silencing Attenuates Myocyte Hypertrophy and Cardiac Fibrosis in MI

**Figure 5:**
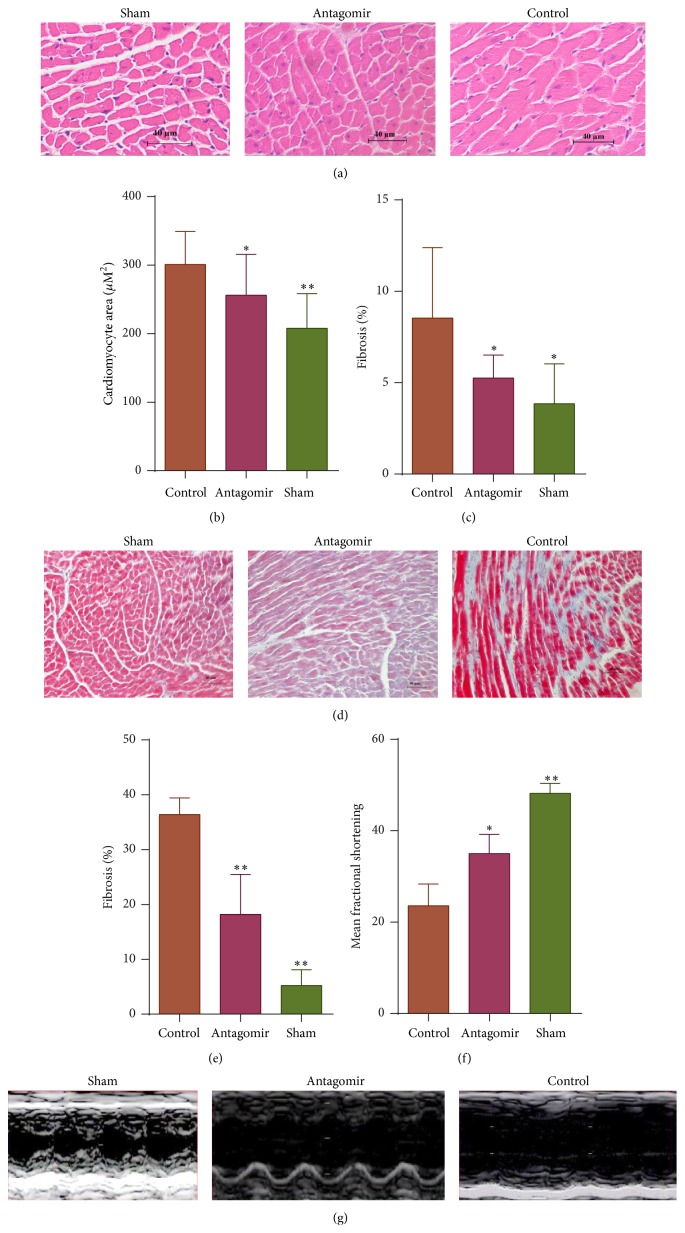
miR-208a silencing attenuates cardiomyocyte hypertrophy and fibrosis and improves cardiac function at 28 days after MI (^*∗*^ *p* < 0.05, ^*∗∗*^ *p* < 0.001). (a) Representative images of hematoxylin and eosin staining indicate a decrease in cardiomyocyte size in antagomir treated mice at 28 days after MI. Scale bar = 40 *µ*m. (b) Graph comparing mean cardiomyocyte sizes at 28 days after MI. Antagomir treatment decreased the mean cardiomyocyte size compared to controls (*n* = 3 per group). (c) Graph showing percentage fibrosis in the noninfarcted area. Antagomir treatment decreased fibrosis in the noninfarcted area compared to controls at 28 days after MI (*n* = 4 per group). (d) Representative images showing miR-208a antagomir decreased fibrosis in the noninfarcted area at 28 days after MI. (e) Bar graph of peri-infarct fibrosis quantification shows reduction in peri-infarct fibrosis in antagomir treatment group at 28 days after MI (*n* = 4 per group). (f) miR-208a silencing improved cardiac function as measured by fractional shortening at 28 days after MI (*n* = 4 per group). (g) Representative echocardiography images showing improved wall motion in antagomir treated group compared to control at day 28 after MI.

At one month after MI, antagomir treatment significantly decreased cardiomyocyte hypertrophy (Figures 5(a) and 5(b)), a known pathological response associated with heart failure and sudden death [19, 20]. Moreover, miR-208a antagomir also decreased percentage fibrosis at day 28 (Figures 5(c), 5(d), and 5(e)). Increased fibrosis is known to reduce cardiac contractility and to contribute to reduction of cardiac function after MI [21], and thus its reduction by antagomir is potentially beneficial. The above results were consistent with previous reports which showed that miR-208a silencing or knockout attenuates cardiac fibrosis and hypertrophy in response to cardiac stress [6, 7].

### 3.5. Therapeutic Silencing of miR-208a Improves Cardiac Function after MI

Aiming to see any beneficial effects of long term silencing of miR-208a on cardiac function after MI, mice received a total of 300 nmols of miR-208a antagomir, and echo was done at 7 and 28 days. Antagomir treatment significantly attenuated cardiac dysfunction after MI (Table 2 and Figures 5(f) and 5(g)).

## 4. Discussion

The current study investigated the role of miR-208a in apoptosis during cardiomyocyte ischemia and the therapeutic potential of miR-208a antagomir in myocardial infarction. Here we reveal for the first time that miR-208a promotes myocyte apoptosis during ischemia and further show that therapeutic administration of miR-208a antagomir attenuates cardiomyocyte apoptosis, hypertrophy, and fibrosis, coupled with improvement in cardiac function after MI.

Following myocardial infarction, nonischemic myocyte apoptosis ensues resulting in further myocardial loss and ventricular dysfunction [2, 19, 20]. Because apoptosis plays a critical role in both mechanical and molecular mechanisms of cardiac dysfunction and remodeling after MI [19, 20], blunting it is an attractive therapeutic target. Our finding that miR-208a antagomir mitigates ischemia induced apoptosis opens a window for its possible therapeutic application in MI. Despite noting no difference in infarct size between the groups, the observed decrease in apoptosis levels could still be a contributor to improved contractile performance, as other studies have shown [2]. Interestingly, some researchers have shown that cardiac miR-208a in human MI is persistently upregulated, with the highest levels observed in patients who died within 24 hours [10]. Thus, targeted silencing of miR-208a in human MI may be a potentially viable and beneficial therapeutic option.

Although miR-208a has been shown to be proproliferative under certain conditions, we demonstrated that it is proapoptotic in the ischemic setting [22, 23]. It is not uncommon for microRNAs or other genes to have opposite effects under different conditions. For example, miR-494 has been shown to have both antiapoptosis and proapoptosis effects under different circumstances [24]. Similarly, p21 (Waf 1), a target of miR-208a, has also been reported to be both anti- and proapoptosis depending on prevailing conditions [25].

To investigate the genetic basis of miR-208a induced apoptosis, we employed custom-built microarrays, which provided an insight into the miR-208a regulated genes associated with apoptosis. There was a noted trend towards downregulation of genes involved in the extrinsic apoptosis pathway and upregulation of those in the intrinsic apoptosis pathway. Among those upregulated was Bax, an essential member in the intrinsic pathway, which was also highlighted by pathway analysis of the differentially expressed apoptosis genes (Figure 3(a)). In MI, Bax was also shown to be downregulated by miR-208a silencing and may thus be playing a role in miR-208a mediated myocyte apoptosis during ischemia. Bax functions by controlling access of upstream apoptotic signals to the mitochondria and together with Bak is essential for intrinsic pathway mediated apoptosis [17, 26]. Given that microRNAs can upregulate gene expression by binding to the promoter regions and target sites in the mRNA or indirectly by downregulating repressors [27–29], we scanned the Bax promoter regions and mRNA for any miR-208a binding sites. Results revealed no predicted miR-208a binding sites both in the gene promoter regions and in mRNA, suggesting that miR-208a may be acting indirectly to alter Bax expression. Our findings only open a window and more studies will be needed to further decipher the mechanisms involved in miR-208a mediated apoptosis.

Upon histological examination, we observed significantly reduced myocyte hypertrophy in the noninfarcted area of the LV following antagomir treatment. Given that heightened cardiomyocyte hypertrophy in the noninfarcted region is associated with remodeling and cardiac dysfunction [19], the noted decrease in myocyte hypertrophy may be a contributor to the observed improvement in cardiac function. Moreover, antagomir therapy attenuated fibrosis, a known contributor to post-MI cardiac dysfunction [30]. Studies have shown that miR-208a functions via upregulation of endoglin to increase myocardial fibrosis, and its silencing downregulates endoglin and reduces type 1 collagen synthesis [31, 32]. The observed decrease in fibrosis provides yet another possible mechanism by which miR-208a antagomir may contribute to improving cardiac function after MI.

Using 2D echocardiography, we demonstrated improved cardiac function at 28 days after MI in animals therapeutically treated with miR-208a antagomir. The observed improvement in cardiac function is likely due to a combination of factors including decrease in apoptosis, hypertrophy, and fibrosis.

To the best of our knowledge, our study is the first to demonstrate that antagomir based miR-208a silencing can attenuate cardiomyocyte apoptosis during ischemia and improve cardiac function after MI. We believe that this will provide a basis for development of therapies targeting miR-208a in MI.

## Figures and Tables

**Table 1:** MicroRNA-208a upregulated or downregulated genes involved in apoptosis.

Downregulated				Upregulated			
Gene	log 2 ratio	Ratio (fold change)	*p* value	Gene	log 2 ratio	Ratio (fold change)	*p* value
STEAP3	−0.7095433	1.6352864	0.0011179	BAX	0.836016276	1.7851141	0.0084057
CRADD	−0.823218	1.7693482	0.0112386	BOK	1.294252566	2.452499	0.0005049
CYLD	−0.6113516	1.5276898	0.0183018	C9	0.74852223	1.680071	0.0019129
CASP8	−0.8671301	1.8240308	0.0016388	ERN1	2.265447191	4.8080343	3.91*E* − 05
COL4A3	−0.8231795	1.7693011	0.0129512	HIPK2	0.593421677	1.508821	1.87*E* − 02
CIDEB	−0.6968248	1.6209334	0.001492	HMOX1	0.644592878	1.5632981	0.0483578
IRF1	−0.6296009	1.5471369	0.0230882	HRAS1	1.335399535	2.5234536	0.0043852
PPP1R13B	−1.029947	2.0419492	0.0006539	WNT7B	1.931190212	3.813697	2.95*E* − 04
DMRT2	−1.0451381	2.0635638	0.0004388				
ERCC2	−0.8137374	1.7577592	7.44*E* − 05				
GSDMA	−0.6040422	1.5199694	0.0236305				
NUDT2	−0.7902432	1.7293659	0.0099716				

**Table 2:** Mean echocardiographic parameters at 7 and 28 days after MI.

	LVIDd	LVIDd	LVIDs	LVIDs	EF	EF	FS	FS
	7 days	28 days	7 days	28 days	7 days	28 days	7 days	28 days
Sham	2.32 ± 0.32	2.82 ± 0.26	1.15 ± 0.15	1.54 ± 0.11	86.6 ± 1.14^*∗*^	82.6 ± 2.3^*∗*^	49.6 ± 1.14^*∗*^	45 ± 2.45^*∗*^
Antagomir	3.68 ± 0.33	3.96 ± 0.41	2.36 ± 0.37	2.59 ± 0.42	72.5 ± 6.19	71.0 ± 5.60^*∗*^	36.0 ± 4.55	35.0 ± 4.2^*∗*^
Control	3.6 ± 0.2	4.07 ± 0.56	2.5 ± 0.26	3.04 ± 0.69	64.8 ± 6.76	53.4 ± 8.79	30.8 ± 4.44	23.6 ± 4.72

LVIDd: left ventricular internal diameter diastole; LVIDs: left ventricular internal diameter systole; EF: ejection fraction; FS: fractional shortening; ^*∗*^ *p* < 0.05 compared to controls.
